# Salivary antibodies induced by BA.4/BA.5-convalescence or bivalent booster Immunoglobulin vaccination protect against novel SARS-COV-2 variants of concern

**DOI:** 10.1128/spectrum.01793-23

**Published:** 2023-08-08

**Authors:** Gabriel Diem, Stefanie Dichtl, Viktoria Zaderer, Cornelia Lass-Flörl, Markus Reindl, Gaia Lupoli, Christopher Dächert, Maximilian Muenchhoff, Alexander Graf, Helmut Blum, Oliver T. Keppler, Doris Wilflingseder, Wilfried Posch

**Affiliations:** 1 Institute of Hygiene and Medical Microbiology, Medical University of Innsbruck, Innsbruck, Austria; 2 Clinical Department of Neurology, Medical University of Innsbruck, Innsbruck, Austria; 3 Max von Pettenkofer Institute and Gene Center, Virology, LMU München, Munich, Germany; 4 Laboratory for Functional Genome Analysis, Gene Center, LMU München, Munich, Germany; National Chung Hsing University, Taichung, Taiwan

**Keywords:** SARS-CoV-2, variants of concern, salivary antibodies, Omicron, vaccination, convalescence

## Abstract

Currently, SARS-CoV-2 Omicron BA.5 subvariants BF.7 and BQ.1.1 are rapidly emerging worldwide. To evaluate the SARS-CoV-2-neutralizing capacity of sera and saliva from triple vaccinated individuals, either boosted with an adapted bivalent COVID-19 vaccine or recovered from BA.4/BA.5 infection, we analyzed the sensitivity of replication-competent SARS-CoV-2 Omicron subvariants BA.4/5, BQ.1.1 and BF.7 to neutralization. Analysis of SARS-CoV-2-specific IgGs and IgAs showed increased serum IgG titers in the vaccinated group, while the serum and salivary IgA levels were comparable. Similar and efficient serum neutralization against the ancestral strain of SARS-CoV-2 and Omicron BA.4/BA.5 was detected in both cohorts, but critically reduced for BQ.1.1 and BF.7. In contrast, salivary neutralization against BA.4/BA.5 was increased in the convalescent compared to the vaccinated group, while salivary neutralizing capacity against BQ.1.1 and BF.7 was comparable in these groups. Further, personalized protective effects studied in a human 3D respiratory model revealed the importance of salivary protection against different Omicron subvariants.

**IMPORTANCE:**

In BA.4/BA.5-convalescent versus vaccinated groups, salivary neutralization capacity increased against SARS-CoV-2 Omicron BA.4/BA.5. In contrast, it neutralized novel Omicron subvariants BQ.1.1 and BF.7 similarly. Salivary protection against various Omicron subvariants was even more evident when tested in a personalized approach using highly differentiated respiratory human 3D models.

## INTRODUCTION

The emergence of new, fast spreading SARS-CoV-2 Omicron subvariants and breakthrough infections give rise to general concern about the efficacy of adapted bivalent COVID-19 vaccines. In less than a year, more than 80% of the population were infected by Omicron subvariants (1, 2). Omicron BA.4/BA.5 remained the dominant variants in many countries for the second half of 2022 and were, therefore, responsible for recent surges of transmissions and COVID-19 (3, 4). SARS-CoV-2 Omicron BA.4 and BA.5 have an identical spike sequence (BA.4/5) and novel subvariants defined as SARS-CoV-2 Omicron BF.7 (BF.7) and Omicron BQ.1.1 (BQ.1.1) emerged later in 2022. The BQ.1.1 subvariant carries the R346T mutation, which is associated with immune escape from vaccine-induced antibodies, along with K444T and N460K substitutions (4 – 6). Some recent articles have shown an extensive escape of these Omicron subvariants using sera from COVID-19 patients who have been vaccinated three or four times (7 – 11). While limited data are currently available comparing the sensitivity of Omicron subvariants to sera from individuals with either a bivalent booster or BA.4/5 breakthrough infections, no data are available for mucosal immunity (6, 7, 12, 13). Here, we analyzed for the first time the sensitivity of replication-competent ancestral SARS-CoV-2 as well as Omicron subvariants BA.4/5, BQ.1.1, and BF.7 to sera and saliva from triple vaccinated individuals, boosted with an adapted bivalent COVID-19 vaccine (4xVac) and three times vaccinated individuals following natural infection with the Omicron BA.4/5 subvariant (3xVac/Conv). We further examined RBD-specific Immunoglobulin G (IgG) in sera and S1-specific IgA levels in serum and saliva from these cohorts.

Especially, the incidence of breakthrough infections in vaccinated individuals during the different Omicron waves highlighted the importance for new additional predictive models to study protection from COVID-19. Although Omicron variants demonstrate lower hospitalization rates and milder disease progression compared to the previous variants of concern (VOCs) such as the Delta variant, protection of high-risk individuals with a poor antibody response after vaccination or a greatly reduced immune function is still of importance. So far, immune protection models were estimated in cohorts over time (14). Nowadays, with changing SARS-CoV-2 variants every few weeks, this approach no longer meets the current requirements for immunity assessment. To address this, we used serum or saliva, respectively, from individuals of both cohorts with high or low antibody titers and tested their protective effects in Omicron BA.4/5, BQ.1.1, or BF.7-infected primary human airway epithelial (HAE) cells grown in air–liquid interface. Overall, in this study we compared neutralizing capacity, antibody titers, and protection of sera and saliva from triple vaccinated individuals, boosted with an adapted bivalent vaccine or recovered from BA.4/5 infection, and, moreover, using a personalized testing approach within a 3D human airway model, we found that salivary immunity, in particular, is essential for protection against novel sub-/variants of concern.

## RESULTS

### SARS-CoV-2-specific antibody responses in serum and saliva

**Fig 1:**
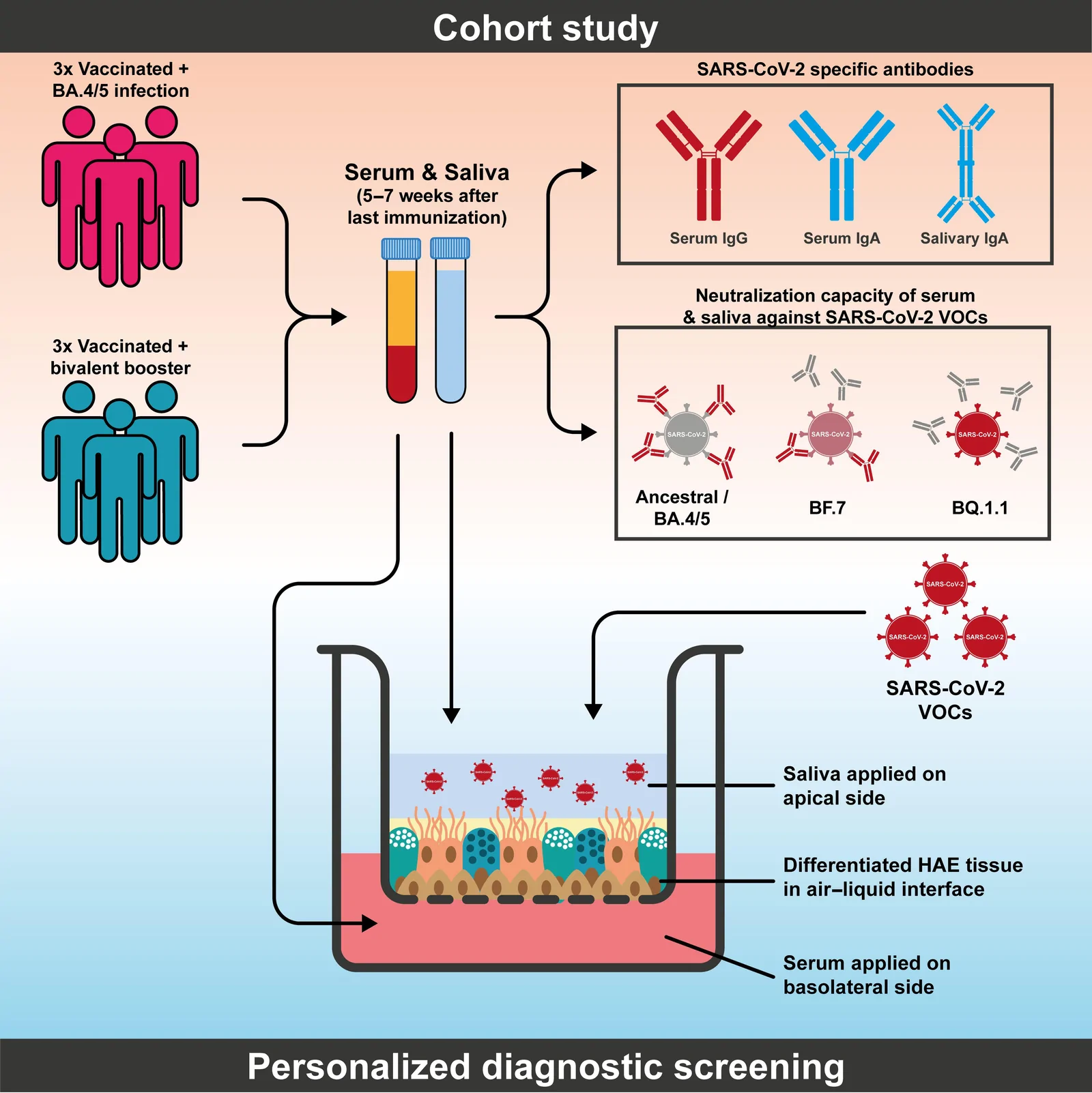
Graphical representation of cohorts and study design. Graphical representation of the chosen cohorts, sample collection and assays performed with an illustration of the personalized diagnostic screening approach where human airway epithelial (HAE) cells are cultured in an air–liquid interface. Patient material can be integrated in this system by adding serum to the basolateral side and saliva on the apical side following infection with SARS-CoV-2 variants of concern to efficiently study the protective effect of serum and/or saliva in a personalized manner and close approximation to physiological conditions.

**Fig 2:**
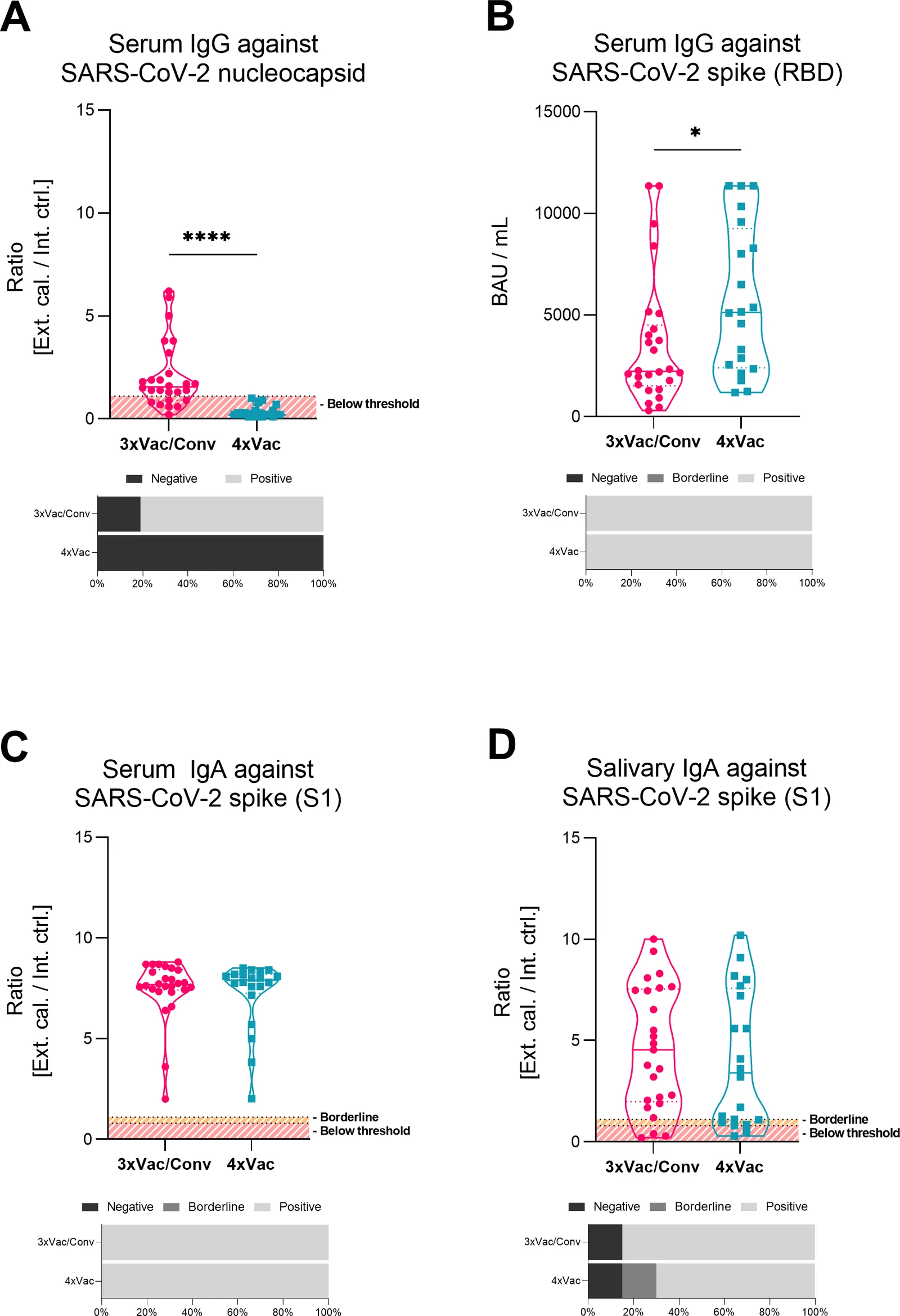
Antibody titers for SARS-CoV-2 nucleocapsid and spike (RBD or S1) induced after BA.4/BA.5 infection or vaccination. Immunoglobulin G (IgG) titers against the SARS-CoV-2 nucleocapsid (**A**), IgG against spike RBD in serum (**B**), and spike S1-specific IgA in serum (**C**) and saliva (**D**) were determined by quantitative or semi-quantitative ELISA. Data from vaccinated and BA.4/5 convalescent (3xVac/Conv, red) and vaccinated and bivalent boostered (4xVac, blue) are shown. Horizontal bar graphs show the percentage of positive, borderline, or negative titers in each cohort. Thresholds for IgG and IgA were set according to manufacturer’s instructions (IgG against spike [RBD]: positive >7.1 binding antibody units per mL [BAU/mL]); nucleocapsid IgG: positive ratio >1.1; IgA against spike [S1]: positive ratio >1.1, borderline ratio 0.9–1.1). Solid lines in violin blots indicate median and dashed lines showing the quartiles. Statistical significance was determined using nonparametric Mann–Whitney test; *P* < 0.05 (*).

Serum and saliva samples were collected 46.5 d (geometric mean) after the last breakthrough infection from the convalescent group and 30.5 d (geometric mean) after receiving the bivalent booster vaccine from the vaccinated group (see Fig. 1 for a graphical depiction of cohorts and study design as well as Table S1 and S2 for a detailed description of vaccine regimen and immunization timepoints). To identify undetected or asymptomatic SARS-CoV-2 infections within the past 6 mo, first, we examined serum IgG titers against SARS-CoV-2 nucleocapsid (N) for the BA.4/5 convalescent (Fig. 2, 3xVac/Conv, pink) and bivalent vaccinated (Fig. 2, 4xVac, blue) cohorts. Although most study participants of both cohorts had breakthrough infection during the Omicron BA.1 and BA.2 wave in Austria, none of the participant from our vaccinated group tested positive for N-specific antibodies (Fig. 2A; Table 1). In contrast, 81% of individuals of our convalescent group tested positive for N-specific IgG (Fig. 2A, pink). Next, we determined serum antibody titers against SARS-CoV-2 receptor binding domain (RBD)-specific IgGs (Fig. 2B; Table 1). Here, all cohorts tested positive, with a geometric mean of 2487.0 binding antibody units per mL (BAU/mL) in the convalescent group, which was significantly higher at 4531.0 BAU/mL in the vaccinated group (Fig. 2B; Table 1). In addition, all subjects tested positive for SARS-CoV-2-specific IgAs against the S1 domain of the viral spike protein in serum, with no significant differences observed between the two groups (Fig. 2C; Table 1). A similar pattern was also shown for S1-specific IgA titers in saliva. Here, convalescent and vaccinated participants displayed positive titers of 85% and 70%, respectively, but no significant differences were found between the cohorts (Fig. 2D; Table 1). To examine if induction of mucosal immunity is dependent on the sheer number of immunizations (infection or vaccination) or also on the virus variant one has been infected with, we further determined IgA titers of triple vaccinated and BA.1- and BA.2-infected individuals. These analyses revealed that individuals vaccinated three times followed by natural infection with Omicron BA.1 or BA.2 had lower salivary IgA titers compared to three times vaccinated and Omicron BA.4/5 convalescent patients (3xVac/BA.4/5), although the sampling time after infection was similar (Fig. S1A and B). Lowest salivary IgA levels were found for triple vaccinated and BA.1-recovered individuals, while similar levels were found for triple vaccinated and BA.2-convalescent and four times vaccinated participants (Fig. S1A). Spearman correlation analysis showed a positive correlation between serum IgG and IgA for both cohorts (Fig. S2A and B) and additionally between serum IgG and salivary IgA in the 4xVac cohort (Fig. S2B). Analysis of serum and salivary antibody titers did not reveal any sex-specific differences between participants from both cohorts (Fig. S3A and B).

**TABLE 1:** List of antibody titers for IgG against SARS-CoV-2 nucleocapsid and spike RBD in serum and IgA against SARS-CoV-2 S1 in serum and saliva^*c*^

Ig isotype (target / domain)	Group	Neutralization % (N / B / P)^*a*^	Geometric mean (95% CI)^*b*^
Serum IgG (nucleocapsid)	3xVac/Conv	19% / 0% / 81%	1.571 (1.147–2.153)
	4xVac	100% / 0% / 0%	0.259 (0.184–0.365)
Serum IgG (spike RBD)	3xVac/Conv	0% / 0% / 100%	2487.0 (1715.0–3195.0)
	4xVac	0% / 0% / 100%	4531.0 (3605.0–6427.0)
Serum IgA (spike S1)	3xVac/Conv	0% / 0% / 100%	7.206 (6.351–8.177)
	4xVac	0% / 0% / 100%	6.902 (5.850–8.142)
Saliva IgA (spike S1)	3xVac/Conv	15% / 0% / 85%	3.178 (2.031–1.521)
	4xVac	15% / 15% / 70%	2.544 (4.973–4.254)

^*a*^ Negative / Borderline / Positive.

^*b*^ IgG (spike RBD) titers: BAU/mL, IgG (nucleocapsid); IgA (spike S1) titers: ratio (ext. control / ext. calibrator).

^*c*^ Percentage of positive, borderline, and negative antibody titers shown as well as the geometric mean with 95% confidence interval of serum IgG against SARS-CoV-2 nucleocapsid (Ratio) and spike RBD (BAU/mL) or IgA against S1 (external control/external calibrator) in serum and saliva.

### Neutralization capacity of serum and saliva against ancestral SARS-CoV-2 and Omicron BA.4/5, BQ.1.1, and BF.7

**Fig 3:**
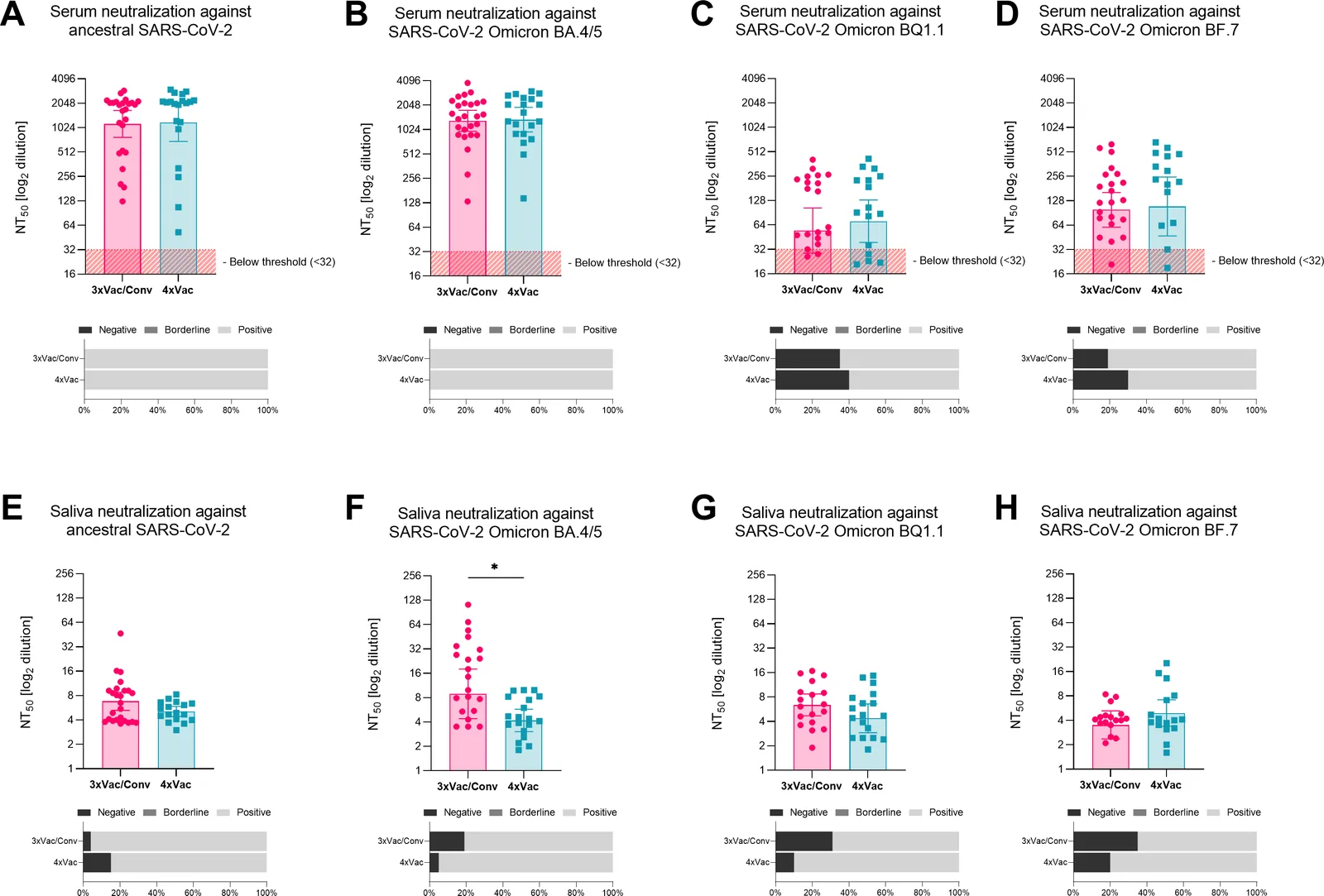
Viral neutralization titers of serum and saliva against ancestral SARS-CoV-2 and Omicron variants BA.4/BA.5, BQ.1.1, and BF.7. Vertical bar graphs show the half-maximal neutralization titers (NT_50_) against ancestral SARS-CoV-2 and Omicron subvariants BA.4/5, BQ.1.1, and BF.7 in serum (**A–D**) and saliva (**E–H**). Data from vaccinated and BA.4/5 convalescent individuals (3xVac/Conv, red) and vaccinated and bivalent boostered individuals (4xVac, blue) are shown. Red lines indicate the defined neutralization threshold. Horizontal bar graphs show the percentage of individuals in each group with positive, borderline, and negative neutralization. For serum, NT_50_ >32 was defined as positive and borderline between NT_50_ 16 and 32 and for saliva, NT_50_ >1 was defined as positive. Data are expressed as geometric mean ± 95% confidence interval. Statistical significance was determined using nonparametric Mann–Whitney test; *P* < 0.05 (*). See also Fig. S5.

In a next step, we assessed the half-maximum neutralization titers (NT_50_) of serum and saliva from vaccinated and recovered individuals against replication competent ancestral SARS-CoV-2 and Omicron subvariants BA.4/5 (BA.4/5), BQ.1.1 (BQ.1.1), and BF.7 (BF.7). All study participants displayed positive serum neutralization against ancestral and BA.4/5, while only 60% and 65% or 70% and 80% neutralized BQ.1.1 and BF.7, respectively (Fig. 3A to D). Highest serum NT_50_ titers were found for 4xVac against ancestral and BA.4/5 (ancestral: 1192.0; BA.4/5: 1356.0), while NT_50_ values for 3xVac/Conv were slightly lower (ancestral: 1139.0; BA.4/5: 1314.0) (Fig. 3A and B; Table 2). Overall, serum neutralizing capacity against the novel subvariants BQ.1.1 and BF.7 was significantly lower compared to ancestral or BA.4/5, but no differences in serum neutralization were detected between the two tested cohorts (Fig. 3C and D; Fig. S4A and B). Lowest NT_50_ titers were found for BQ.1.1 compared to BF.7, and neutralizing capacity compared to the BA.4/5 decreased up to 24-fold for 3xVac/Conv and 19-fold for 4xVac (Fig. 3C and D; Fig. S5; Table 2). Although not significant, comparing serum and salivary Igs against NT_50_ titers in general, Spearman correlations were higher for Igs against serum NT_50_ in the vaccinated cohort while correlations of Igs against salivary NT_50_ were mostly elevated in the convalescent group (Fig. S2).

**TABLE 2:** List of NT_50_ values of serum and saliva against ancestral SARS-CoV-2 or Omicron subvariants BA.4/5, BQ.1.1, and BF.7^*c*^

	Virus variant	Group	Neutralization % (N / B / P)^*a*^	Geometric mean (95% CI)^*b*^
Serum	Ancestral	3xVac/Conv	0% / 0% / 100%	1139.0 (776.8–1669.0)
		4xVac	0% / 0% / 100%	1192.0 (692.6–2052.0)
	BA.4/5	3xVac/Conv	0% / 0% / 100%	1314.0 (976.6–1768.0)
		4xVac	0% / 0% / 100%	1356.0 (956.3–1922.0)
	BQ.1.1	3xVac/Conv	35% / 0% / 65%	54.5 (28.7–103.6)
		4xVac	40% / 0% / 60%	71.1 (38.9–129.8)
	BF.7	3xVac/Conv	19% / 0% / 81%	99.0 (60.5–162.0)
		4xVac	30% / 0% / 70%	108.7 (47.1–250.9)
Saliva	Ancestral	3xVac/Conv	4% / 0% / 96%	6.812 (5.261–8.820)
		4xVac	15% / 0% / 85%	5.105 (4.419–5.896)
	BA.4/5	3xVac/Conv	19% / 0% / 81%	8.899 (4.389–18.04)
		4xVac	5% / 0% / 95%	4.187 (3.042–5.762)
	BQ.1.1	3xVac/Conv	31% / 0% / 69%	6.396 (4.70–8.704)
		4xVac	10% / 0% / 90%	4.373 (2.904–6.585)
	BF.7	3xVac/Conv	35% / 0% / 65%	3.512 (2.356–5.235)
		4xVac	20% / 0% / 80%	4.924 (3.379–7.175)

^*a*^ N, negative; B, borderline; P, positive.

^*b*^ NT_50_ titers: reciprocal dilutions.

^*c*^ Percentage of positive, borderline, and negative neutralizers as well as geometric mean of the measured NT_50_ values from 3xVac/Conv and 4xVa groups with 95% confidence interval are presented.

Investigating the saliva samples of 3xVac/Conv and 4xVac, we found a reduced percentage of positive neutralizers against all tested viruses (Fig. 3E through H. In contrast to serum neutralization, not all participants were able to neutralize the ancestral strain of SARS-CoV-2 or the BA.4/5 subvariant (Fig. 3E and F; Fig. S4; Table 2). Interestingly, a significant higher saliva neutralization titer against BA.4/5 was observed in the convalescent (3xVac/Conv) compared to the vaccinated (4xVac) cohort, but this was not found against the ancestral strain (Fig. 3E and F; Fig. S4; Table 2). In contrast to ancestral SARS-CoV-2 and BA.4/5, both cohorts demonstrated reduced saliva NT_50_ titers against the new Omicron subvariants, with the lowest neutralizing capacity detected against BF.7 (Fig. 3G and H; Fig S4; Table 2). In fact, this reduced neutralizing power of BF.7 in comparison to BA.4/5 in the convalescent group was shown to be significant, while no significant differences in neutralization were found within the vaccinated group (Fig. S4C and D). Additionally, similar results in serum and salivary neutralization against all tested virus variants were found for male and female participants with no significant differences observed. The results were independent of previous breakthrough infection during the BA.1 and BA.2 wave in Austria (Fig. S3C and D, S5).

### Personalized protection analysis of serum and saliva against Omicron BA.4/5, BQ.1.1, and BF.7

**Fig 4:**
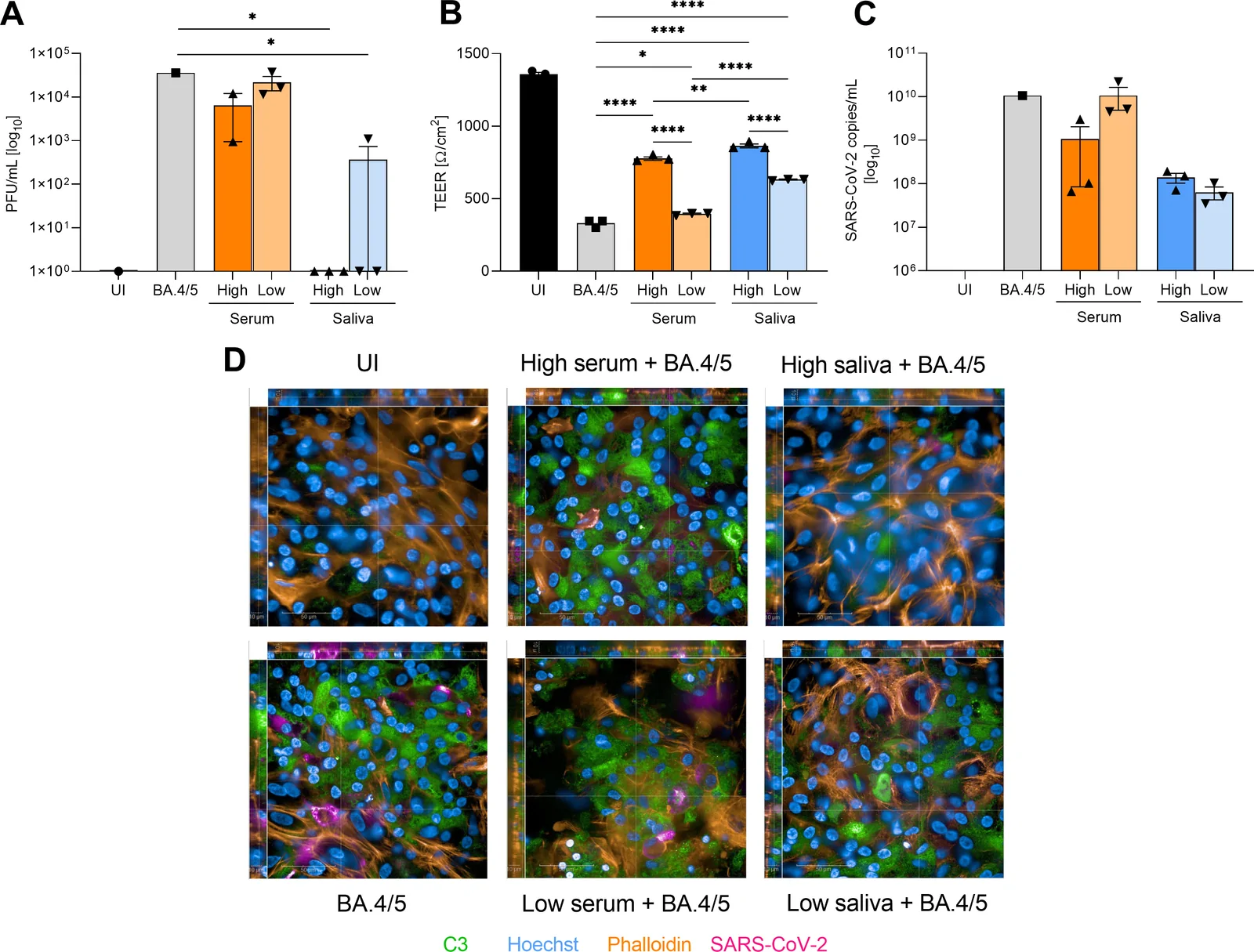
Personalized protection analysis of serum and saliva against Omicron BA.4/5. After 72 h of infection, plaque assays of supernatants (**A**), TEER (**B**), and viral RNA (**C**) were measured from uninfected (UI), BA.4/5-infected human airway epithelium (HAE) ±serum, or saliva from three individuals from the high (*n* = 3) and low group (*n* = 3), respectively. Representative images of XYZ stacks are shown, C3 in green, SARS-CoV-2 N in pink, nuclei (Hoechst) in blue, and actin (phalloidin) in orange (**D**). Scale bars represent 50 µm and 10 µm as indicated. Statistically significant differences were determined by one-way ANOVA with Tukey correction. Data are presented as mean ± SEM; *P* < 0.05 (*); *P* < 0.01 (**); *P* < 0.0001 (****).

To investigate, if vaccinated and convalescent individuals from our cohorts were also protected against Omicron BA.4/5, BQ.1.1, and BF.7 subvariants, we designed an experimental setting to test the efficacy of serum- or saliva-mediated protection from SARS-CoV-2 infections in a human 3D respiratory model. For this, primary HAE cells were grown in air–liquid interface (ALI) for 30 d and heat-inactivated serum or saliva samples from individuals with high (HT) or low antibody titers (LT) were applied (Table S3). To evaluate protection between HT and LT groups, three individuals with either high (IgG in serum >3000 BAU/mL and IgA in saliva >4) or low antibody titers were chosen independently of their vaccination regimen (3xVac/Conv, 4xVac) (Table S3). Serum and saliva samples were obtained from the same individual. Such ALI cultures of primary HAE-cells display a healthy and highly differentiated, pseudostratified, mucus-producing, ciliated respiratory tissue model and have been established and standardized in our lab as described (15). In addition to serum, we also chose to test the protection of saliva, since it represents the body fluid, which is in closest contact to the lower respiratory tract. We mimicked the infection route by adding saliva to the apical side or serum to the basolateral side before HAE cells were infected with clinical isolates of SARS-CoV-2 BA.4/5, BF.7, or BQ.1.1. After 72 h of infection, cells were analyzed using plaque assay, transepithelial electrical resistance (TEER), quantitative RT-PCR, and confocal image analyses for SARS-CoV-2 infection. Additionally, complement component 3 (C3) signal was measured as a marker for inflammation. While TEER measurement is used as an indicator for tissue integrity, plaque assays allow quantification of active viral particles in supernatants of the HAE cells (Table 3). Uninfected conditions served as negative controls, while infected cells without any addition of serum or saliva were used as positive controls in all experiments. Interestingly, plaque assays of BA.4/5-infected cells revealed that the addition of serum from the HT and LT group was not able to significantly neutralize infectious viral particles (Fig. 4A, orange bars). In contrast, salivary samples of the HT group reduced viral titers in all individuals to zero, which was also the case for two out of three saliva samples from the LT group (Fig. 4A, blue bars and Table 3). Similar results were analyzed measuring tissue integrity by TEER. Although the addition of serum from the HT group could retain the TEER values compared to BA.4/5-infected cells, this effect was significantly enhanced when saliva samples of the HT group were applied in ALI cultures prior to BA.4/5 infection (Fig. 4B, Table 3). Of note, addition of serum or saliva to uninfected HAE cells resulted in hardly any differences of the tissue integrity (Fig. S6) and thereby, highlighting that serum or saliva does not display any toxic effects on the HAE cells. Serum and, in particular, saliva samples from the LT group could also maintain tissue integrity upon viral infection, but these effects were significantly lower compared to the results obtained from the HT group (Fig. 4B). Analyzing viral load by absolute SARS-CoV-2 copynumber quantification revealed results comparable to the plaque assay. Here, the HT and LT serum groups were not able to reduce viral particles in HAE cells, while both saliva groups reduced the viral load (Fig. 4C, Table 3). Furthermore, SARS-CoV-2 infection and inflammation measured by C3 induction were evaluated via confocal image analysis (Fig. 4D). C3 activation in SARS-CoV-2-infected HAE cells was previously shown to initiate a highly inflammatory microenvironment, resulting in different grades of tissue damage (15, 16). We analyzed SARS-CoV-2 BA.4/5 positive cells in the presence or absence of HT and LT serum or saliva. While the addition of HT serum reduced the viral signal, this effect was only slightly reduced in the presence of LT serum compared to the BA.4/5-infected control (Fig. 4D, pink signal, center panels). The addition of LT saliva further decreased the virus signal, while highest reduction was detected only by HT saliva samples (Fig. 4D, pink signal, right panels). Of note, only HT saliva samples could reduce the inflammatory C3 expression in addition to the reduced infection (Fig. 4D, green signal).

**TABLE 3:** List of mean values measured for plaque forming units, TEER, and SARS-CoV-2 copy number from samples tested on HAE cells in air–liquid interface and challenged with SARS-CoV-2 variants BA.4/5, BQ.1.1, and BF.7^*d*^

VOC	Condition	PFU/ml^*a*^	TEER^*b*^	SARS-CoV-2 copies^*c*^
BA.4/5	Untreated	0.0 (0.0)	1357.0 (14.5)	0.0 (0.0)
	BA.4/5	36000.0 (0.0)	330.0 (15.0)	1.07 × 10^10^ (0.0)
	Serum high	6475.0 (5525.0)	776.7 (12.0)	1.06 × 10^9^ (9.77 × 10^8^)
	Serum low	21833.0 (7965.0)	395.0 (5.0)	1.06 × 10^10^ (5.73 × 10^9^)
	Saliva high	1.0 (0.0)	863.3 (13.3)	1.39 × 10^8^ (3.55 × 10^7^)
	Saliva low	367.3 (366.3)	631.7 (3.3)	6.36 × 10^7^ (2.09 × 10^7^)
BQ.1.1	Untreated	0.0 (0.0)	1703.0 (8.8)	0.0 (0.0)
	BQ.1.1	43000.0 (0.0)	406.7 (3.3)	1.77 × 10^9^ (0.0)
	Serum high	1060.0 (1045.0)	533.3 (3.3)	2.36 × 10^8^ (1.40 × 10^8^)
	Serum low	4117.0 (671.0)	415.0 (12.6)	9.45 × 10^8^ (2.19 × 10^8^)
	Saliva high	134.0 (133.0)	853.3 (6.7)	1.82 × 10^7^ (5.95 × 10^6^)
	Saliva low	9302.0 (7662.0)	320.0 (2.9)	5.15 × 10^8^ (2.08 × 10^8^)
BF.7	Untreated	0.0 (0.0)	1703.0 (8.8)	0.0 (0.0)
	BF.7	75500.0 (0.0)	385.0 (10.4)	8.10 × 10^8^ (0.0)
	Serum high	21.0 (19.0)	671.7 (15.9)	2.37 × 10^7^ (1.06 × 10^7^)
	Serum low	28500.0 (7365.0)	378.3 (1.7)	5.47 × 10^8^ (1.22 × 10^8^)
	Saliva high	1.0 (0.0)	975.0 (2.9)	9.16 × 10^5^ (3.81 × 10^5^)
	Saliva low	5167.0 (3417.0)	633.3 (14.5)	5.47 × 10^7^ (2.39 × 10^7^)

^*a*^ Data are presented as mean plaque forming units per mL (± SEM).

^*b*^ Data are presented as mean Ω/cm^2^ (±SEM).

^*c*^ The mean copynumber of the nucleocapsid gene (±SEM).

^*d*^ Summarized results of PFU/mL, TEER analysis, and viral copynumber determination presented as mean ± SEM.

**Fig 5:**
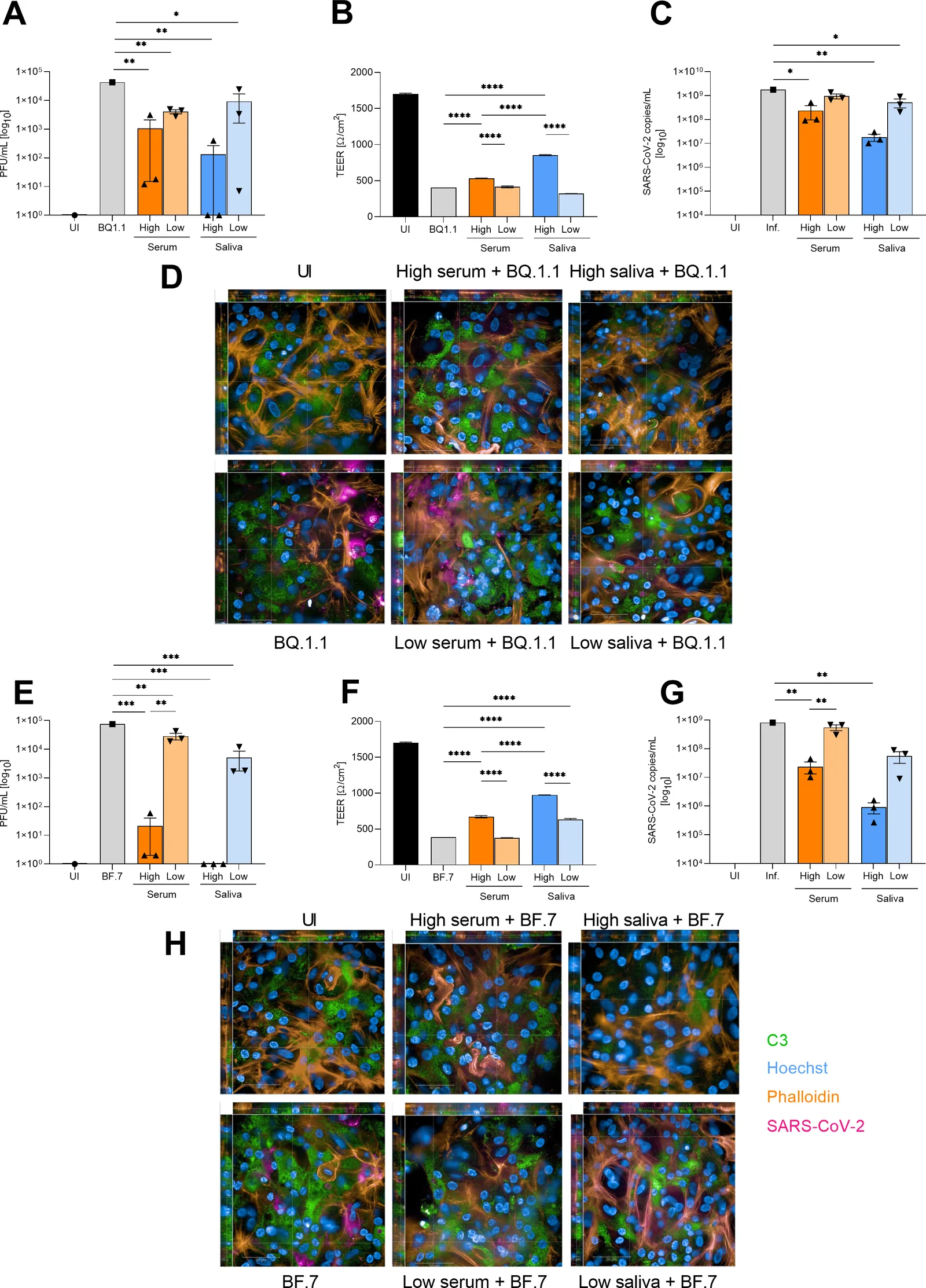
Personalized protection analysis of serum and saliva against Omicron subvariants BQ.1.1 and BF.7. After 72 h of infection, plaque assays of supernatants (**A**), TEER (**B**), and viral RNA (**C**) was measured from uninfected (UI), BQ.1.1-infected HAE ± serum, or saliva from three individuals from the high (*n* = 3) and low group (*n* = 3), respectively. Representative pictures of XYZ stacks are shown: C3 in green, SARS-CoV-2 N in pink, nuclei (Hoechst) in blue, actin (phalloidin) in orange (**D**). Scale bars represent 50 µm and 10 µm as indicated. Plaque assays of supernatants (**E**), TEER measurement (**F**), and determination of SARS-CoV-2 copies (**G**) were also performed on BF.7-infected HAE cells ± serum or saliva from three individuals from the high and low group, respectively. Representative images show XYZ stack of these conditions (**H**). Statistically significant differences were determined by one-way ANOVA with Tukey correction. Data are presented as mean ± SEM; *P* < 0.05 (*); *P* < 0.01 (**); *P* < 0.001 (***); *P* < 0.0001 (****). See also Fig. S6 and Table S3.

Finally, we analyzed the protective capacity of HT and LT samples against new SARS-CoV-2 VOCs in HAE cells. Similar to BA.4/5 infection, we added serum or saliva to ALI cultures prior to infection with Omicron BQ.1.1 or BF.7 subvariants either basolaterally (serum) or apically (saliva). Adding serum from the HT group resulted in significantly decreased viral titers compared to untreated BQ.1.1 (Fig. 5A) or BF.7-infected cells (Fig. 5E). These plaque assays demonstrated that the addition of LT serum samples also resulted in a significant, but less distinct, decrease of the viral titers. As demonstrated before using BA.4/5, the presence of saliva from the HT group completely blocked BQ.1.1 or BF.7 infection in two out of three or three out of three individuals, respectively, while the LT saliva group inhibited the viral titers to lower levels (Fig. 5A and E). These findings were also supported by analyzing the tissue integrity (Table 3). The addition of HT serum and, in particular, HT saliva significantly maintained elevated TEER values; this effect was less prominent with the LT serum and saliva samples (Fig. 5B and F). Moreover, the analysis of BQ.1.1 and BF.7 viral load and infection by absolute quantification using RT-PCR and image analyses confirmed the highest protective capacity by saliva samples of the HT group (Fig. 5C, D, G, and H). Additionally, LT saliva displayed a diminished virus signal, but only the HT saliva group abolished the virus and inflammatory expression completely (Fig. 5C, D, G, and H, pink and green signal, right panel). To exclude donor-specific differences of HAE cells causing these observed antiviral effects, we further used uninfected cells as well as cells infected with BQ.1.1 or BF.7 from an additional donor in combination with serum and saliva from the HT group and measured the tissue integrity with similar results (Fig. S7A and B). The addition of serum or saliva to HAE cells of the second donor confirmed the elevated protection against BF.7 in contrast to BQ.1.1 infection (Fig. S7C), as demonstrated here also by neutralization assays (Fig. 3).

## DISCUSSION

In this study, we compared the humoral immune responses of individuals, either triple vaccinated against SARS-CoV-2 following natural infection with Omicron BA.4/5 or triple vaccinated individuals and boosted with a BA.4/5 adapted, bivalent vaccine. First, we examined serum IgG titers against the viral N to exclude current, undiagnosed, or asymptomatic BA.4/5 infections in the vaccinated cohort. We could confirm that all participants of the vaccinated group were negative for N-specific antibodies, although most study participants of both cohorts had breakthrough infections during the Omicron BA.1 and BA.2 wave in Austria in early 2022. Next, we measured the serum SARS-CoV-2-RBD-specific IgG levels and detected significantly higher antibody titers in the vaccinated compared to the convalescent group, which is in line with previous findings observing the tendentious increased S1 IgG levels (17). The detection of serum and salivary IgAs displayed comparable titers between the two groups. We and others previously demonstrated that SARS-CoV-2-specific IgAs are detected in serum after intramuscular immunization of COVID-19 vaccines (18, 19). Overall, in this study, we observed a trend of higher salivary IgA levels in convalescent compared to vaccinated individuals. Results from this and previous studies demonstrated that not only the numbers of immunization events play an important role in induction of salivary IgA but also the virus variant (18, 20).

Additionally, we determined the half maximum neutralization capacity (NT_50_) against replication-competent SARS-CoV-2 using both serum and saliva, to investigate neutralization against ancestral SARS-CoV-2, BA.4/5, and the novel VOCs BQ.1.1 and BF.7 in our cohorts. Both groups showed comparable serum neutralization against all SARS-CoV-2 variants. In accordance with the literature, we also found a critically reduced neutralization capacity of serum against BQ.1.1 and BF.7 compared to BA.4/5 or the ancestral strain, resulting in a 12–21-fold reduction, respectively (7, 21, 22). Moreover, we observed generally higher serum neutralization capacity against BF.7 than BQ.1.1 in both groups, which is also in agreement with previous observations (7, 21, 22). Our results further highlight that an infection with BA.4/5 as well as receiving a bivalent booster vaccine does not mount a strong neutralizing capacity in serum against new Omicron subvariants. In addition to serum, we also chose to test the protection of saliva, since it represents the body fluid, which is in closest contact to the lower respiratory tract and amongst the first in contact with the virus. The respiratory tract naturally moves mucus from the lungs into the throat, where it mixes with saliva (23). Therefore, saliva is in direct contact with SARS-CoV-2 during the infection route and can be collected in a noninvasive manner. Here we found that salivary neutralization against ancestral SARS-CoV-2, BQ.1.1, and BF.7 was comparable between vaccinated and recovered individuals. In contrast, convalescent individuals exhibited a stronger salivary neutralizing capacity against BA.4/5 than vaccinated individuals, suggesting that natural infection may induce mucosal antibody-mediated protection more efficiently than the bivalent booster vaccine (18). Salivary protection against ancestral, BA.4/5, BQ.1.1, and BF.7 was comparable in the bivalent vaccinated group, while for convalescent individuals, salivary neutralization was highest against BA.4/5 and even significantly higher compared to BF.7. However, 20–35% of convalescent individuals were negative for salivary neutralization against Omicron variants, showing that natural infection more frequently fails to induce protective neutralization capacity in saliva. Of note, no sex-specific differences between males and females were detected in serum and salivary neutralization and also previous breakthrough infection during the BA.1 and BA.2 wave in Austria had no effect on these results.

Personalized protection analysis of vaccinated and convalescent participants with high antibody titers also revealed that especially the addition of saliva lead to increased protection against BA.4/5 infection in HAE cells. Testing the antiviral efficacy of serum in our 3D respiratory model considerably reduced viral infection and enhanced tissue integrity, but to a lesser extent than HT saliva. In general, saliva offered a better protection against BA.4/5 infection compared to serum. At the same time, HT samples showed stronger antiviral efficacy than LT samples.

Investigating the protection of novel SARS-CoV-2 variants, BQ.1.1 and BF.7, in the 3D model demonstrated a declining antiviral effect compared to BA.4/5, which was most prominent against BQ.1.1. These data are in accordance to the NT_50_ titers demonstrated here and confirm a diminished antiviral protection of our convalescent or vaccinated cohorts against novel VOCs supporting previous studies (7, 17). Additionally, our data provide new evidence that the presence of salivary, but not serum antibodies, could dampen excessive inflammation by reduced complement activation and anaphylatoxin production. In previous studies, it was shown that SARS-CoV-2 infections strongly induce C3-enriched foci within the pseudostratified epithelia, resulting in significantly elevated secreted C3a levels, and initiating a highly inflammatory microenvironment, causing severe tissue damage (15, 16, 24). The differences in complement activation could also explain the observed changes in the tissue integrity between the high and low saliva group, although the viral titers are comparable. The personalized protection analysis demonstrates a sophisticated but not complete model of the respiratory infection route. In this study, the HAE cells were not supplemented with immune cells, which clearly play an important role in the outcome of an infection. Further, the addition of the saliva on the apical side mimics the natural infection but cannot exclude a direct neutralizing effect of the saliva to the virus, due to the fact that both were added on the same side in contrast to the serum. This assay tries to simulate the infection occurring in the respiratory tract with a complex and highly developed human model with some methodical restrictions. Limitations of this study include the small cohort size, the unknown effect of previous exposure to SARS-CoV-2, and comparison of the vaccines at a single time point. Therefore, future studies analyzing long-term effects of lasting immunity are essential. The mucosal immunity in vaccinated people or individuals with hybrid-immunity (3xVac/Conv) over time is highly controversial. Some studies report an increase of salivary IgA after 8 mo from the BNT162b2-booster vaccination (25), while others show a decline in nasal IgA response 9 mo after hospitalization for COVID-19 and a minimal impact of subsequent vaccination (26). Therefore, the results with the newly developed COVID-19 vaccine candidate NDV-HXP-S are of great interest and may lighten this controversial field. This vaccine candidate can also be administered intranasally and, therefore, show for the first time if an intranasal vaccine candidate is comparably effective to an intramuscular one (27).

Overall, our data emphasize that three times vaccinated individuals, who received the bivalent booster, as well as SARS-COV-2-vaccinated individuals following natural infection with the Omicron subvariants BA.4 or BA.5, show comparable serum and salivary IgA values and neutralization capacities against the new sublineages of SARS-CoV-2. Due to the overall low neutralization titers against BQ.1.1 and BF.7, an updated vaccine strategy might be beneficial. Symptoms and hospitalization rates of BF.7- and BQ.1.1-infected individuals seem to be comparable to other Omicron subvariants, although cohort studies are still missing (28). Recent studies investigating neutralization efficiency against BQ.1.1 and XBB in individuals, who received a bivalent booster vaccine, clearly demonstrated a substantial neutralization escape by these novel variants (6, 11, 12). Thus, these findings and our data emphasize reconsidering of future vaccine booster strategies from intramuscular to an oral or intranasal approach, which could provide a more potent and protective immunity as demonstrated in animal experiments (29, 30). By establishing a personalized protection assay in a human 3D respiratory model, we could discover the importance of serum- and saliva-mediated protection in close approximation to physiological conditions. This personalized diagnostic test also revealed the magnitude of salivary protection, which remained effective across different SARS-CoV-2 variants, including novel VOCs.

## MATERIALS AND METHODS

### Human samples

In this study, serum and saliva samples of 46 individuals were collected and divided into two groups. The first group was vaccinated three times against SARS-CoV-2 following natural infection with the Omicron subvariant BA.4/5 (3xVac/Conv; *n* = 26; Table S1). The second group was vaccinated three times following Pfizer’s BA.4 and BA.5 adapted bivalent booster vaccine (4xVac; *n* = 20; Table S2). For the first three vaccines, all individuals received either ChAdOx1 (AstraZeneca), mRNA-1273 (Moderna), or BNT162 (Biontech/Pfizer). Seven out of 26 individuals (26.9%) in the 3xVac/Conv group and 14 out of 20 individuals (70%) in the 4xVac group were previously infected either with the ancestral strain of SARS-CoV-2, Delta, or Omicron subvariants BA.1 or BA.2. All these individuals, including those who had COVID-19 infection, were diagnosed via PCR and showed mild disease severity, which did not require any treatment or hospitalization. The virus variant of these individuals was validated via mutant-specific PCR or inferred by date of diagnosis (first positive PCR test) and prevalence of the current variant in the specific region. The geometric mean sampling day after last immunization (vaccination or infection) for 3xVac/Conv and 4xVac was 46.5 d and 30.0 d, respectively.

### Viruses and virus propagation

Ancestral SARS-CoV-2 virus was obtained from the BEI Resource repository and propagated according to the manufacturer’s instructions. Clinical specimen for SARS-CoV-2 Omicron (B.1.1.529) BA.4 and BA.5 were isolated from SARS-CoV-2-PCR-positive swabs and variants confirmed by mutant-specific PCR (Ethics statement, ECS1166/2020). High-titer, replication-competent virus stocks of BF.7 (GISAID EPI_ISL_15825638) and BQ1.1 (GISAID EPI_ISL_15812431) were expanded from clinical isolates and characterized by variant-specific PCR (31) and whole genome sequencing, in principle, as reported (32 – 34).

### Antibody titer determination in serum and saliva against nucleocapsid and spike S1 and receptor binding domain (RBD) from ancestral SARS-CoV-2

Serum from vaccinated or COVID-19 convalescent participants was obtained from blood samples in serum collection tubes by centrifugation at 300 g for 5 min, carefully collected and stored at −80°C until use. Saliva samples were collected using saliva collection tubes (Salivette). As suggested by the manufacturer, the liquid phase was obtained after centrifugation at 4,000 g for 5 min and stored at −80°C until use. Sera and saliva were analyzed with SARS-CoV-2 IgG II Quant Assay (Abbott, USA). The chemiluminescent microparticle immunoassay (CMIA) SARS-CoV-2-IgG-II-Quant-Assay was performed in order to assess anti-SARS-CoV-2 IgGs against RBD. CMIA results were calculated to BAU/mL according to manufacturer instructions and the cut-off value for positive results was defined at 7.1 BAU/mL. Serum was also tested for SARS-CoV-2 IgG against nucleocapsid and IgA against S1 using commercially available ELISA assays (Anti-SARS-CoV-2-ELISA IgA, Euroimmun, Lübeck, Germany). IgG titers against nucleocapsid in sera and IgA antibody titers against spike (S1) in sera and saliva were analyzed via anti-SARS-CoV-2 IgG against nucleocapsid and anti-SARS-CoV-2 IgA against spike S1. Results are shown as a ratio (external control/external calibrator), with a ratio ≥1.1 defined as positive, as suggested by the manufacturer. No significant differences were found in antibody titers against SARS-CoV-2 nucleocapsid, spike (RBD), and spike (S1) between male and female individuals (Fig. S3A and B).

### Immunofluorescence neutralization assay

VeroE6-TMPRSS2-ACE2 cells (2 × 10^4^) were seeded in a 96-well plate with DMEM high glucose medium supplemented with 10% FCS, 1% L-Glutamine, and 1% Penicillin/Streptomycin. and incubated overnight at 37°C and 5% CO_2_. On the following day, heat-inactivated serum and saliva samples were serially diluted from 1:8 to 1:4096 or 1:4 to 1:512, respectively. Dilutions were incubated with ancestral SARS-CoV-2, BA.4/5, BQ.1.1, or BF.7 variant (5 × 10^2^ PFU/mL) for 1 h at 37°C, and subsequently dilutions were used as inoculum and transferred to VeroE6-TMPRSS2-ACE2 cells for 1 h at 37°C and 5% CO_2_. After incubation, inoculum was aspirated, cells washed with D-PBS, and incubated in DMEM supplemented with 1.5% FCS, 1% L-Glutamine, and 1% Penicillin/Streptomycin at 37°C and 5% CO_2_. After 16 h, medium was removed and cells fixed in 4% Formalin for 30 min at room temperature. After fixation, cells were permeabilized for 20 min according to the manufacturer’s instructions. The same buffer was used during immunofluorescence staining using in combination with a primary antibody against SARS-CoV-2 nucleocapsid and a fluorescently labeled secondary antibody. Cells were washed twice with D-PBS before imaging, followed by automated spot count and quality control using an ImmunoSpot analyzer and ImmunoSpot Software v5.0.9.15. Half-maximum neutralization titers (NT_50_) were calculated using normalized four-parameter nonlinear regression model in GraphPad Prism v.9. A positive neutralization threshold was defined as 1:32 for serum and 1:1 for saliva.

### Human airway epithelia (HAE)

Human airway epithelia (HAE) cells (Lonza; cat# CC-2540S) are available in our laboratory and routinely cultured in air–liquid interface (ALI) as described (15, 35). Briefly, cells were cultured in a T75 flask using Pneuma Cult Ex Plus medium (Stemcell; cat# 05040) supplemented with hydrocortisone (Stemcell; cat# 07980) for 2–4 d until they reached 80% confluence. The cells were dissociated with an Animal Component-Free Cell Dissociation Kit (Stemcell; cat# 05426) and seeded onto collagen-coated 0.33 cm^2^ porous (0.4 µm) polyester membrane inserts with a seeding density of 1  ×  10^5^ cells per Transwell. The cells were grown to near confluence in submerged culture for 2–3 d in Pneuma Cult Ex Plus medium supplemented with hydrocortisone. Cultures were maintained in a humidified atmosphere with 5% CO2 at 37°C and then transferred to ALI culture. The epithelium was expanded and differentiated using Pneuma Cult ALI medium (Stemcell; cat# 05021) supplemented with hydrocortisone and 0.2% heparin solution (Provitro; cat#0863). The number of days in development was designated relative to initiation of ALI culture, corresponding to day 0.

### Virus infection of HAE cells

HAE cells were cultured in ALI for 30 d. One day before infection, 10% heat-inactivated serum was added to the basolateral side or 50 µL heat-inactivated saliva to the apical side, respectively. The cells were infected with an MOI of 0.01 of clinical specimen of SARS-CoV-2 apically. After 24 h of infection, all cells (uninfected, infected, with or without serum or saliva) were washed to perform the TEER measurement. Therefore, the apical administered saliva was also washed away. The cells were harvested on day 3 post infection (3dpi).

### TEER measurement

TEER values were measured using an EVOM volt ohm meter with STX-2 chopstick electrodes. Measurements on cells in ALI culture were taken at 3dpi. For measurements, 100 µL medium was added to the apical side. Cells were allowed to equilibrate before TEER was measured.

### Immunofluorescence staining and imaging

After infection, cells were fixed with 4% paraformaldehyde. Intracellular staining was performed using 1× Intracellular Staining Permeabilization Wash Buffer. Antibodies to detect nuclei, actin (phalloidin), and complement C3 were used. Intracellular SARS-CoV-2 was detected using Alexa594-labeled SARS-CoV-2 antibodies against the viral nucleocapsid (N). Cells were thoroughly washed following the staining procedure using the permeabilization buffer, sterile-filtered with D-PBS, and finally mounted in Mowiol. The Operetta CLS system was used to image the samples. Analysis was performed using the Harmony software 4.9.

### Real-time RT-PCR for absolute quantification of SARS-CoV-2

SARS-CoV-2 RNA was extracted using FavorPrep Viral RNA Mini Kit, according to manufacturer’s instructions. SARS-CoV-2 levels were quantified using the primer-probe combinations designed by the Centers for Disease Control and Prevention (CDC) 2019-nCoV Real-Time RT-PCR Diagnostic Panel. Sequences specific to two distinct regions of the nucleocapsid (N) gene, N1 and N2, and for the detection of a human housekeeping gene, ribonuclease P published on the CDC website (https://www.cdc.gov/coronavirus/2019-ncov/lab/rt-pcr-panel-primer-probes.html), were used. Single target assays of all three targets were performed in combination with the Luna Universal Probe One-Step RT-qPCR Kit. For absolute quantification using the standard curve method, SARS-CoV-2 RNA was obtained as a PCR standard control from the National Institute for Biological Standards and Control (Ridge, UK). The SARS-CoV-2 RNA was used in 10-fold dilutions according to the manufacturer’s instructions. All runs were performed on a Bio-Rad CFX 96 instrument and analyzed by the Bio-Rad CFX Maestro 1.1 software.

### Virus plaque assay

Plaque assay was modified from reference 19. VeroE6-TMPRSS2-ACE2 (9 × 10^4^) were seeded in a 48-well plate in DMEM high glucose supplemented with 10% fetal calf serum, 1% L-glutamine, and 1% penicillin/streptomycin, and incubated overnight at 37°C and 5% CO_2_. On the next day, supernatant from the HAE cells was serially diluted and used as inoculum on VeroE6-TMPRSS2-ACE2 cells for 1 h at 37°C. After inoculation, supernatant was removed and culture medium containing 1.5% carboxymethylcellulose was added. Cells were incubated for 3 d at 37°C and 5% CO_2_ before plaque visualization and counting. For this, cells were washed and fixed with 10% neutral buffered formalin for 1 h at room temperature. Fixation was followed by staining using 0.5% (w/v) crystal violet solution for 15 min in the dark at room temperature.

### Statistical analysis

Statistical analysis was performed using GraphPad Prism v.9. Significances of antibody titers and NT_50_ levels were determined using nonparametric Mann–Whitney test, and one-way ANOVA with Tukey correction was used to assess significant differences of HAE cell experiments.

## Data Availability

Any additional information required to reanalyze the data reported in this work paper is available from the lead contact upon request.

## SUPPLEMENTAL MATERIAL

Supplemental information spectrum.01793-23-s0001.docx Supplemental tables and figures.

ASM does not own the copyrights to Supplemental Material that may be linked to, or accessed through, an article. The authors have granted ASM a non-exclusive, world-wide license to publish the Supplemental Material files. Please contact the corresponding author directly for reuse.
