# Supplementary material for: Salivary antibodies induced by BA.4/BA.5-convalescence or bivalent booster Immunoglobulin vaccination protect against novel SARS-COV-2 variants of concern
Source: Microbiol Spectr. 2023 Aug 8;11(5):e01793-23. doi: 10.1128/spectrum.01793-23 (PMC10581068; doi:10.1128/spectrum.01793-23)
Supplement: Supplemental information — Supplemental tables and figures. [file spectrum.01793-23-s0001.docx]

**Table S1: Three times vaccinated and SARS-CoV-2 BA.4/5 recovered individuals (3xVac/Conv)**

| ID | Sex | Age | Vaccination scheme^a^ | COVID-19 recovered | Variants of previous infections | Date of diagnosis with BA.4/5 | Days between 3^rd^ vaccination and infection | Days after last immunization |
| --- | --- | --- | --- | --- | --- | --- | --- | --- |
| C1 | M | 31 | PPP | Yes | BA.2 / BA.4/5 | 01.08.2022 | 262 | 73 |
| C2 | F | 48 | AAP | Yes | BA.4/5 | 27.08.2022 | 280 | 47 |
| C3 | M | 50 | AAP | Yes | BA.4/5 | 02.09.2022 | 286 | 45 |
| C4 | F | 43 | PPP | Yes | BA.4/5 | 30.08.2022 | 329 | 44 |
| C5 | M | 37 | MMM | Yes | BA.4/5 | 29.08.2022 | 251 | 49 |
| C6 | F | 33 | PPP | Yes | BA.4/5 | 29.08.2022 | 251 | 57 |
| C7 | F | 50 | APP | Yes | BA.4/5 | 29.09.2022 | 314 | 36 |
| C8 | M | 55 | MMP | Yes | BA.4/5 | 29.09.2022 | 286 | 36 |
| C9 | M | 50 | AAP | Yes | BA.1 / BA.4/5 | 09.10.2022 | 262 | 29 |
| C10 | F | 20 | PPP | Yes | BA.4/5 | 05.10.2022 | 239 | 33 |
| C11 | M | 37 | APP | Yes | BA.1 / BA.4/5 | 26.07.2022 | 241 | 85 |
| C12 | M | 50 | PPP | Yes | BA.4/5 | 23.09.2022 | 315 | 34 |
| C13 | F | 50 | AAP | Yes | BA.4/5 | 19.09.2022 | 304 | 38 |
| C14 | F | 52 | AAP | Yes | BA.4/5 | 10.10.2022 | 314 | 32 |
| C15 | F | 35 | PPP | Yes | BA.4/5 | 04.10.2022 | 144 | 37 |
| C16 | M | 49 | PPP | Yes | BA.4/5 | 04.10.2022 | 144 | 37 |
| C17 | F | 59 | AAP | Yes | BA.4/5 | 05.10.2022 | 353 | 37 |
| C18 | M | 32 | AAP | Yes | BA.4/5 | 25.07.2022 | 253 | 86 |
| C19 | F | 46 | AAM | Yes | BA.4/5 | 27.09.2022 | 330 | 45 |
| C20 | F | 46 | AAP | Yes | Ancestral / BA.4/5 | 28.05.2022 | 208 | 167 |
| C21 | F | 29 | PPP | Yes | BA.4/5 | 20.09.2022 | 243 | 52 |
| C22 | F | 62 | PPP | Yes | Delta / BA.4/5 | 16.10.2022 | 257 | 30 |
| C23 | M | 27 | MMP | Yes | BA.4/5 | 12.10.2022 | 306 | 35 |
| C24 | M | 51 | AAP | Yes | BA.4/5 | 06.10.2022 | 330 | 40 |
| C25 | F | 45 | AAM | Yes | BA.1 / BA.4/5 | 03.10.2022 | 299 | 43 |
| C26 | M | 32 | APP | Yes | BA.2 / BA.4/5 | 18.08.2022 | 252 | 89 |
| n = 26 | | | | | | | | |
| Age, Geometric mean (95% CI): 41.6 (37.3 – 46.5) | | | | | | | | |
| Sex ratio (M/F): 46.2% / 53.8% | | | | | | | | |
| Days between 3rd vaccination and infection, Geometric mean (95% CI): 265.6 (242.9 – 290.4) | | | | | | | | |
| Days between last immunization and sampling, Geometric mean (95% CI): 46.5 (39.4 – 54.9) | | | | | | | | |
| ^a^ A: ChAdOx1 (AstraZeneca), M: mRNA-1273 (Moderna), P: BNT162 (Biontech Pfizer) | | | | | | | | |

**Table S2: Individuals, three times vaccinated and boostered with BA.4 / BA.5 adapted bivalent booster vaccine (4xVac)**

| ID | Sex | Age | Vaccination scheme^a^ | COVID-19 recovered | Variants of previous infections | Date of last vaccination | Days between last infection and 4^th^ vaccination | Days after last immunization |
| --- | --- | --- | --- | --- | --- | --- | --- | --- |
| V1 | F | 27 | PPPP+ | Yes | BA.1 | 01.09.2022 | 211 | 54 |
| V2 | F | 33 | MMPP+ | Yes | Delta | 29.09.2022 | 312 | 36 |
| V3 | M | 35 | AAPP+ | Yes | BA.1 | 29.09.2022 | 262 | 39 |
| V4 | M | 30 | AAPP+ | Yes | BA.1 | 12.10.2022 | 201 | 26 |
| V5 | F | 32 | PPPP+ | Yes | BA.1 | 15.10.2022 | 249 | 27 |
| V6 | F | 51 | AAPP+ | Yes | BA.2 | 19.10.2022 | 225 | 19 |
| V7 | M | 57 | AAPP+ | No | - | 06.10.2022 | - | 28 |
| V8 | F | 28 | AAPP+ | No | - | 05.10.2022 | - | 29 |
| V9 | F | 32 | AAMP+ | No | - | 30.09.2022 | - | 41 |
| V10 | M | 28 | AAPP+ | Yes | Delta | 30.09.2022 | 330 | 41 |
| V11 | F | 40 | APPP+ | Yes | BA.1 | 22.10.2022 | 254 | 24 |
| V12 | M | 40 | AAPP+ | Yes | BA.2 | 18.10.2022 | 145 | 30 |
| V13 | M | 59 | PPPP+ | No | - | 21.10.2022 | - | 27 |
| V14 | F | 28 | PPMP+ | Yes | BA.2 | 31.10.2022 | 222 | 21 |
| V15 | M | 37 | PPPP+ | Yes | BA.2 | 17.10.2022 | 207 | 36 |
| V16 | F | 39 | PPPP+ | No | - | 05.10.2022 | - | 48 |
| V17 | F | 41 | PPMP+ | Yes | BA.2 | 03.11.2022 | 265 | 20 |
| V18 | F | 36 | PPMP+ | Yes | BA.2 | 26.10.2022 | 223 | 28 |
| V19 | F | 58 | PPMP+ | No | - | 22.10.2022 | - | 32 |
| V20 | M | 60 | AAPP+ | Yes | BA.2 | 02.11.2022 | 246 | 27 |
| n = 20 | | | | | | | | |
| Age, Geometric mean (95% CI): 38.2 (33.6 – 43.3) | | | | | | | | |
| Sex ratio (M/F): 37.0% / 63.0% | | | | | | | | |
| Days between last infection and 4^th^ vaccination, Geometric mean (95% CI): 235.1 (209.3 – 264.1) | | | | | | | | |
| Days between last immunization and sampling, Geometric mean (95% CI): 30.5 (26.7 – 34.8) | | | | | | | | |
| ^a^ A: ChAdOx1 (AstraZeneca), M: mRNA-1273 (Moderna), P: BNT162 (Biontech Pfizer), P+: BA.4 and BA.5 adapted bivalent booster vaccine (Biontech Pfizer) | | | | | | | | |

**Table S3: Antibody and NT_50_ titers of low and high neutralizers used for HAE experiments**

Table shows the antibody titers for IgG against Nucleocapsid, IgG against spike (RBD) and IgA against spike (S1) in serum as well as IgA against spike (S1) in saliva. In addition, NT_50_ values against all tested SARS-CoV-2 VOCs are shown.

| Group | | High titer | | | Low titer | | | | |
| --- | --- | --- | --- | --- | --- | --- | --- | --- | --- |
| ID | | #1 | #2 | #3 | #1 | #2 | | | #3 |
| Cohort | | 3xVac/Conv | 4xVac | 4xVac | 3xVac/Conv | 4xVac | | | 4xVac |
| Serum | IgG (N) | 1.4 | 0.1^a^ | 0.2^a^ | 5.9 | | 0.2^a^ | 0.1^a^ | |
|  | IgG (RBD) | 3759.1 | 11360 | 11360 | 465.5 | | 1242.5 | 2359.3 | |
|  | IgA (S1) | 7.9 | 8.2 | 8.1 | 7.34 | | 2.0 | 7.8 | |
| Saliva | IgA (S1) | 7.5 | 4.1 | 5.6 | 3.78 | | 1.1 | 0.8^a^ | |
| Serum NT_50_^b^ | Ancestral | 315.4 | 2153.1 | 2130.3 | 1291.9 | | 52.7 | 1231.9 | |
|  | BA.4/5 | 2161.0 | 1149.8 | 2805.9 | 829.4 | | 906.8 | 701.5 | |
|  | BQ.1.1 | 48.0 | 13.0^a^ | 91.0 | 26.0 | | 28.0 | 36.0 | |
|  | BF.7 | 191.0 | 203.0 | 451.0 | 75.0 | | 10.0^a^ | 12.0^a^ | |
| Saliva NT_50_^b^ | Ancestral | 15.7 | 4.7 | 5.8 | 3.7 | | 0.0^a^ | 4.9 | |
|  | BA.4/5 | 68.9 | 9.9 | 3.7 | 3.5 | | 0.7^a^ | 1.8 | |
|  | BQ.1.1 | 9.0 | 3.9 | 2.4 | 3.1 | | 6.7 | 4.4 | |
|  | BF.7 | 6.9 | 4.7 | 15.3 | 0.0^a^ | | 1.6 | 0.0^a^ | |
| ^a^ Values below threshold  ^b^ NT_50_ titers: reciprocal dilutions | | | | | | | | | |


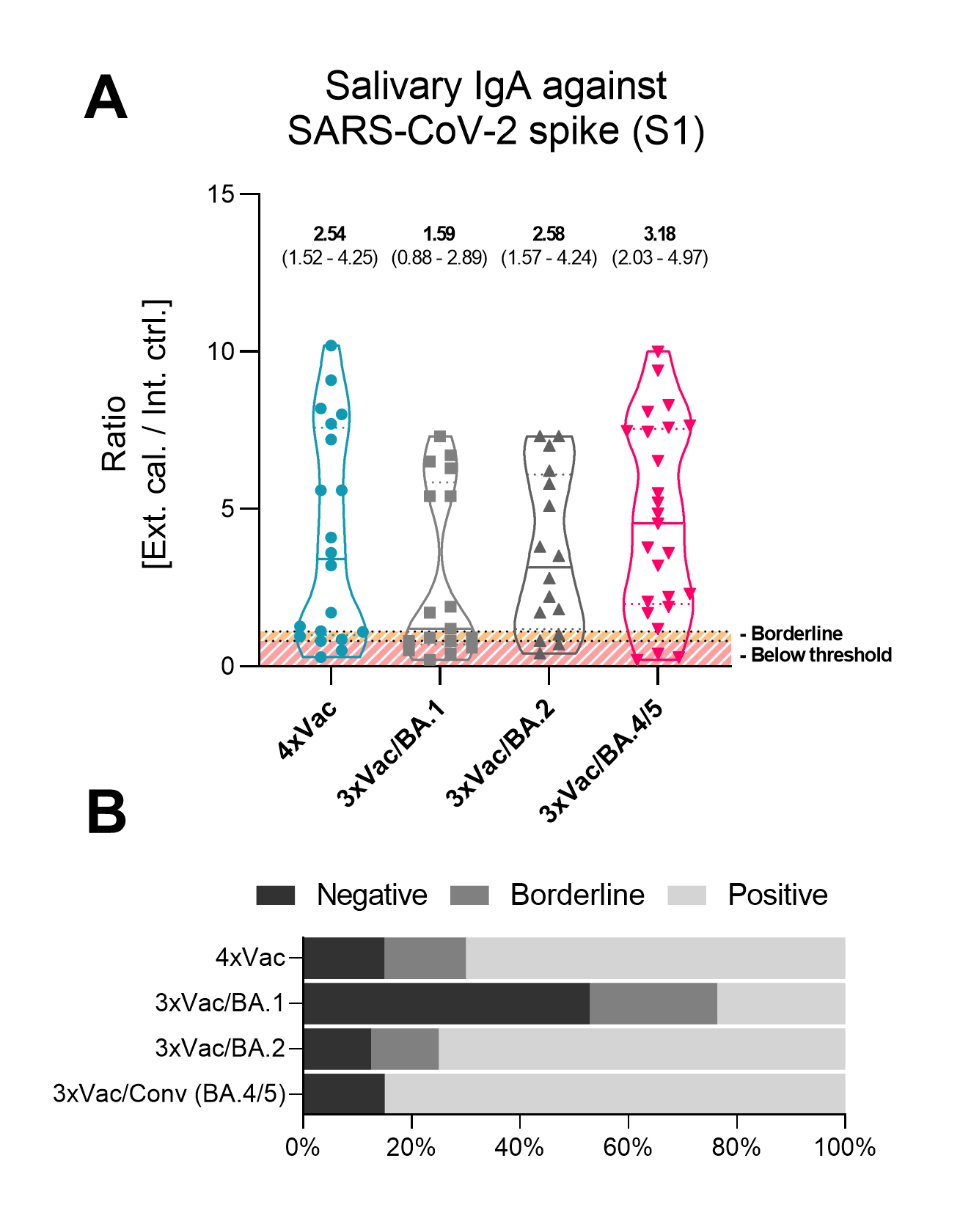


**Figure S1: Differences in viral neutralization of serum and saliva against SARS-CoV-2 variants**

**(A)** The graph shows IgA titers against the SARS-CoV-2 spike S1 in saliva of individuals which were vaccinated three times following booster vaccination with the BA.4/5 adapted booster vaccine. In addition, we included individuals, which were three times vaccinated following an infection with either SARS-COV-2 omicron BA.1, BA.2 or BA.4/5. Data from BA.1 and BA.2 convalescent individuals were used from a previous study (unpublished). For the BA.1 recovered group (n=22), the geometric mean age was 36.5 years with a 95% CI of 13.1 and a sex ratio of 50% / 50% (M/F). Days between last vaccination and infection was 61.5 (13.1) and days after last immunization and sampling was 48.2 (22.6). For the BA.2 convalescent group (n=22), the geometric mean age and 95% CI was 33.4 (4.8), sex ratio of 36% / 64% (M/F), days between last vaccination and infection 114.6 (14.2) and days after last immunization and sampling was 67.4 (10.7). Solid lines in violin blots indicate median and dashed lines showing the quartiles. The values above each violin blot indicates the geometric mean (± 95% CI). **(B)** Horizontal bar graphs show the percentage of positive, borderline, or negative titers in each cohort. Thresholds for IgA were set according to manufacturer's instructions (positive ratio >1.1, borderline ratio 0.9 - 1.1) Statistical analysis was performed using non-parametric Kruskal-Wallis test with Dunn’s multiple comparison.


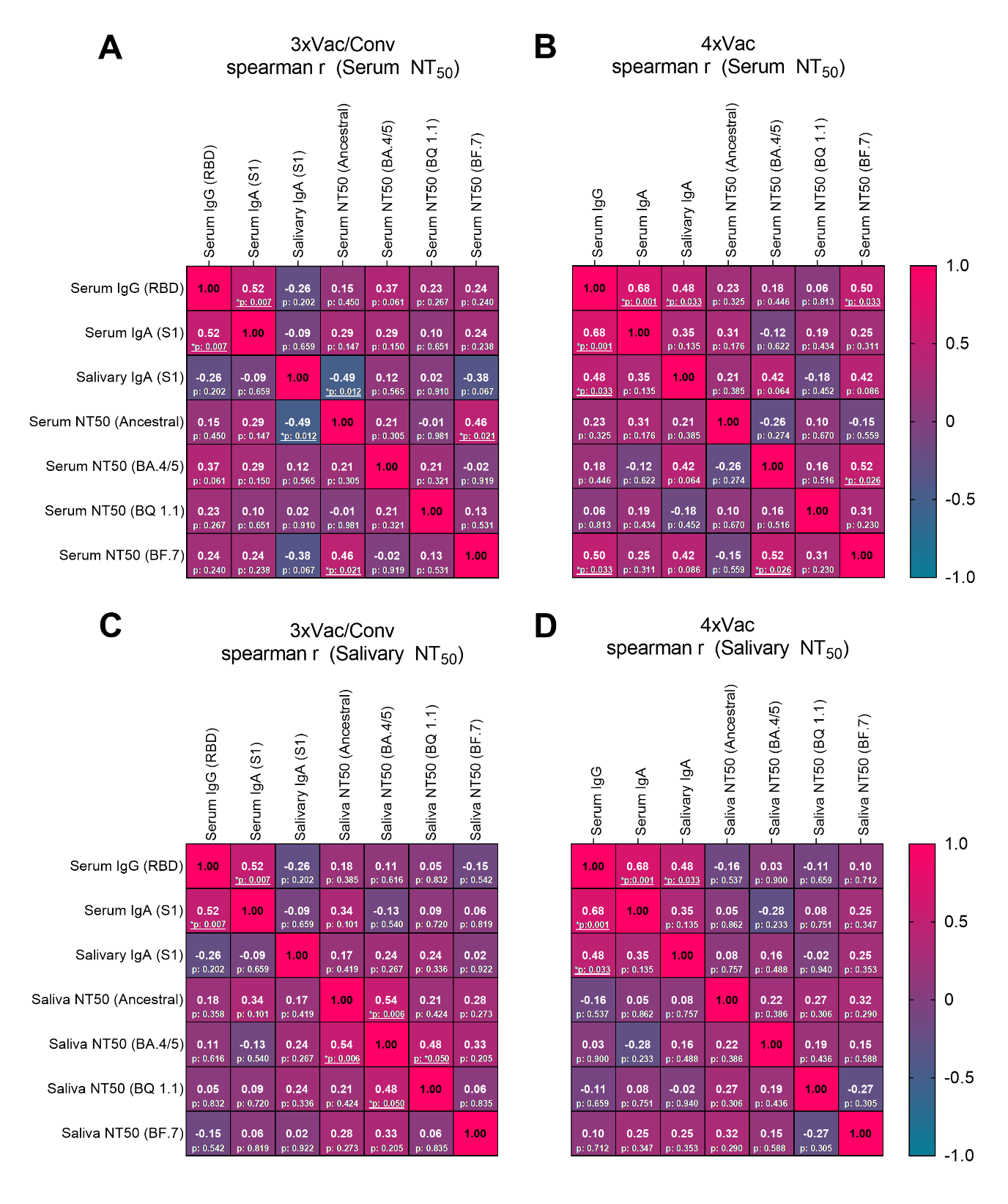


**Figure S2: Spearman correlation matrix of antibody titers and serum/saliva neutralization**

Heatmaps show a non-parametric, two-tailed spearman correlation matrix of antibody titers and NT_50_ values of the tested variants in serum **(A-B)** and saliva **(C-D)** of vaccinated and recovered groups. Numbers represent the Spearman r (upper value) and p (lower value). Underlined p-values indicate significance with p<0.05.


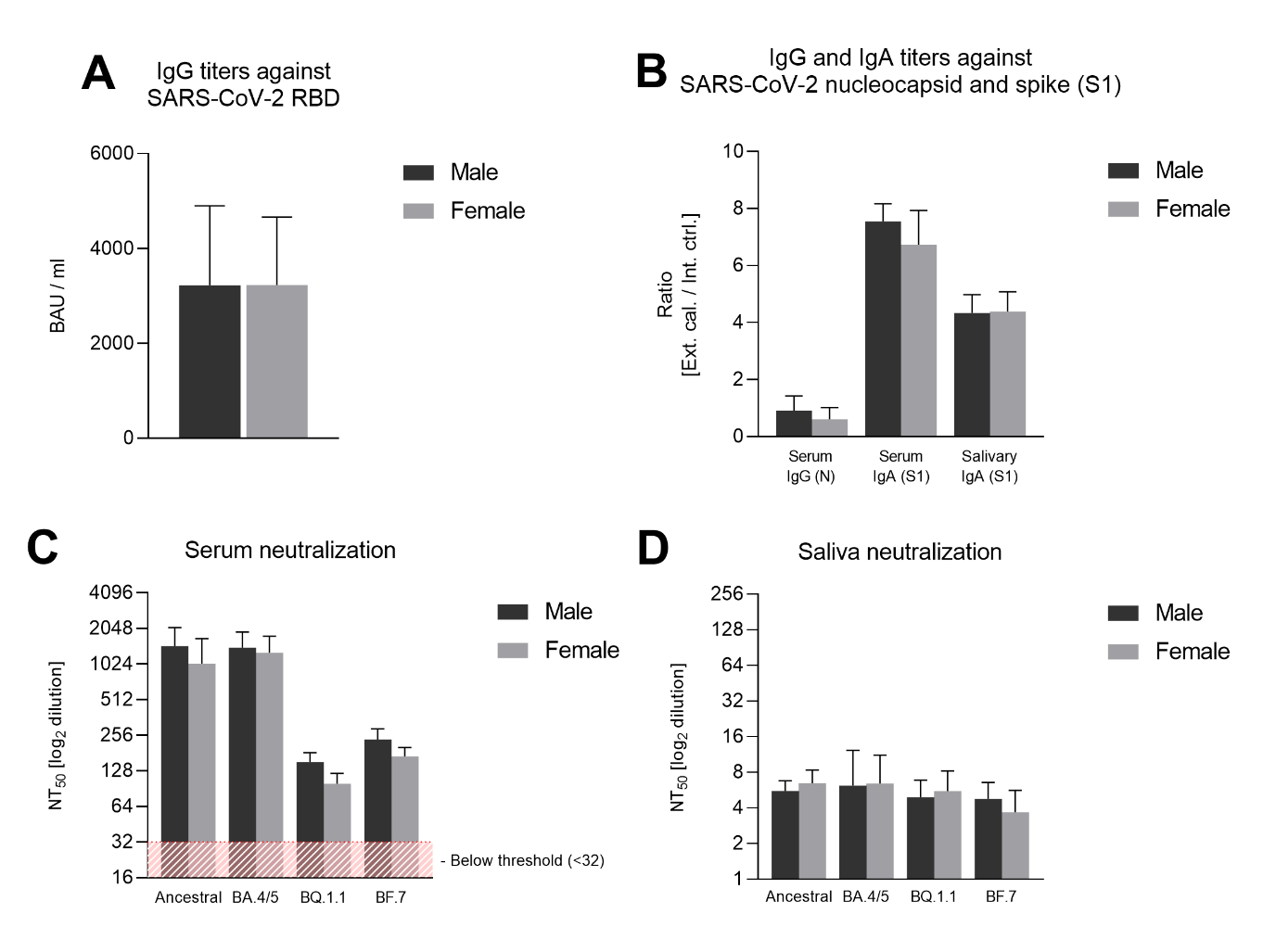
**Figure S3: Sex specific differences in antibody titers and viral neutralization in serum and saliva against SARS-CoV-2 variants**

Graphs show the IgG titers of male and female participants against SARS-CoV-2 spike RBD **(A)**, IgG against nucleocapsid, IgA against spike (S1) in serum and saliva **(B)** as well as half-maximum neutralization against ancestral SARS-CoV-2, BA.4/5, BQ.1.1 and BF.7 in serum **(C)** or saliva **(D)**. Data are expressed as geometric mean ± 95% confidence interval. Statistical significance was determined using non-parametric Mann-Whitney test for IgG against spike (RBD) or Kruskal-Wallis test with Dunn’s multiple comparison for IgG titers against nucleocapsid, IgA titers against spike (S1) and NT_50_ of serum and saliva; p: 0.0021 (**); p: 0.0002 (***); p<0.0001 (****).


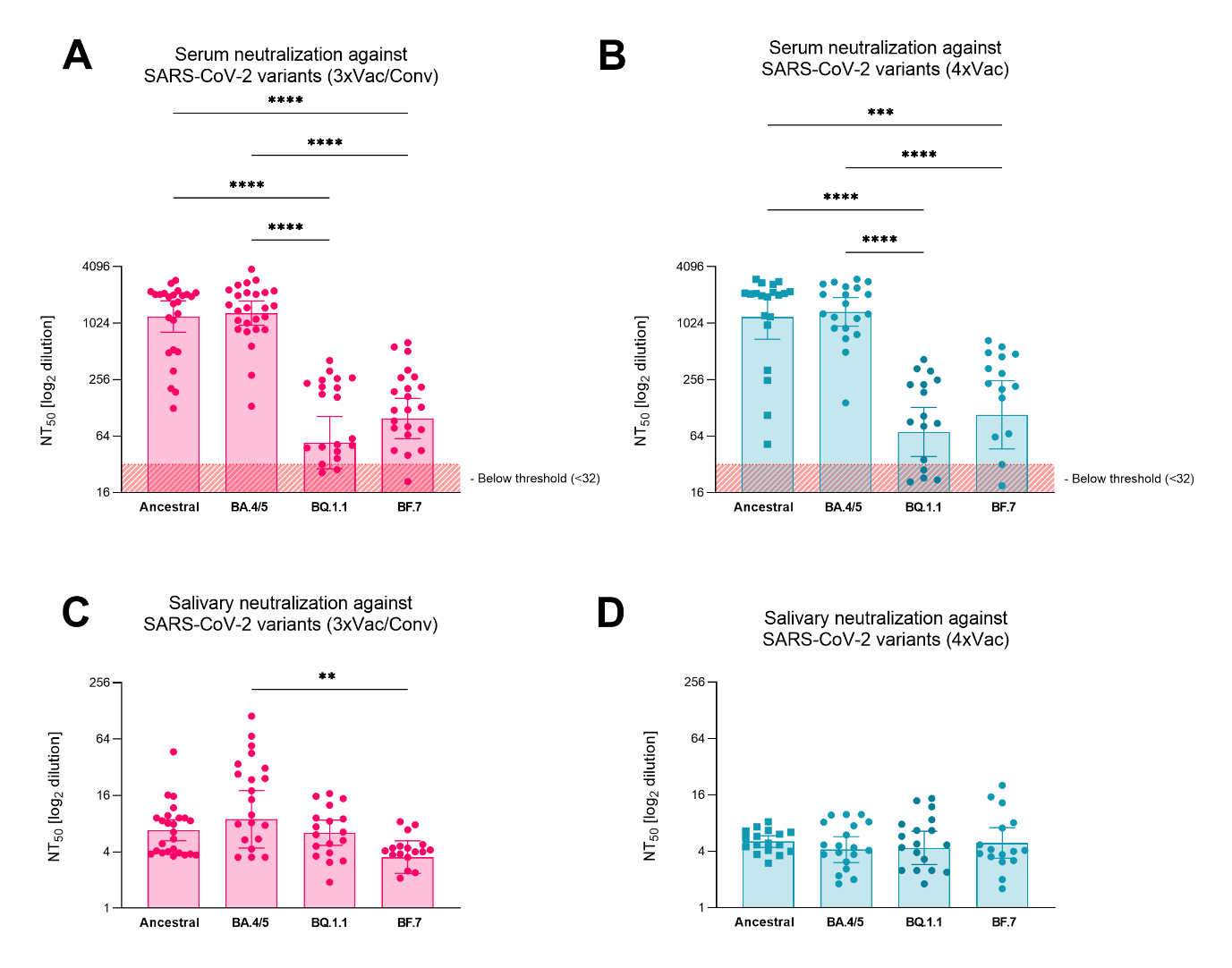


**Figure S4: Differences in viral neutralization of serum and saliva against SARS-CoV-2 variants**

Graphs show the half-maximal neutralization titers against ancestral SARS-CoV-2 and omicron subvariants BA.4/5, BQ.1.1, and BF.7 in thrice vaccinated and BA.4/5 convalescent **(A-B)** or thrice vaccinated and bivalent boostered individuals **(C-D)**. Red lines indicate the defined neutralization threshold. For serum, NT_50_>32 was defined as positive and borderline between NT_50_ 16 and 32 and for saliva, NT_50_>1 was defined as positive. Data are expressed as geometric mean ± 95% confidence interval. Statistical significance was determined using non-parametric Kruskal-Wallis test with Dunn’s multiple comparison; p: 0.0021 (**); p: 0.0002 (***); p<0.0001 (****).


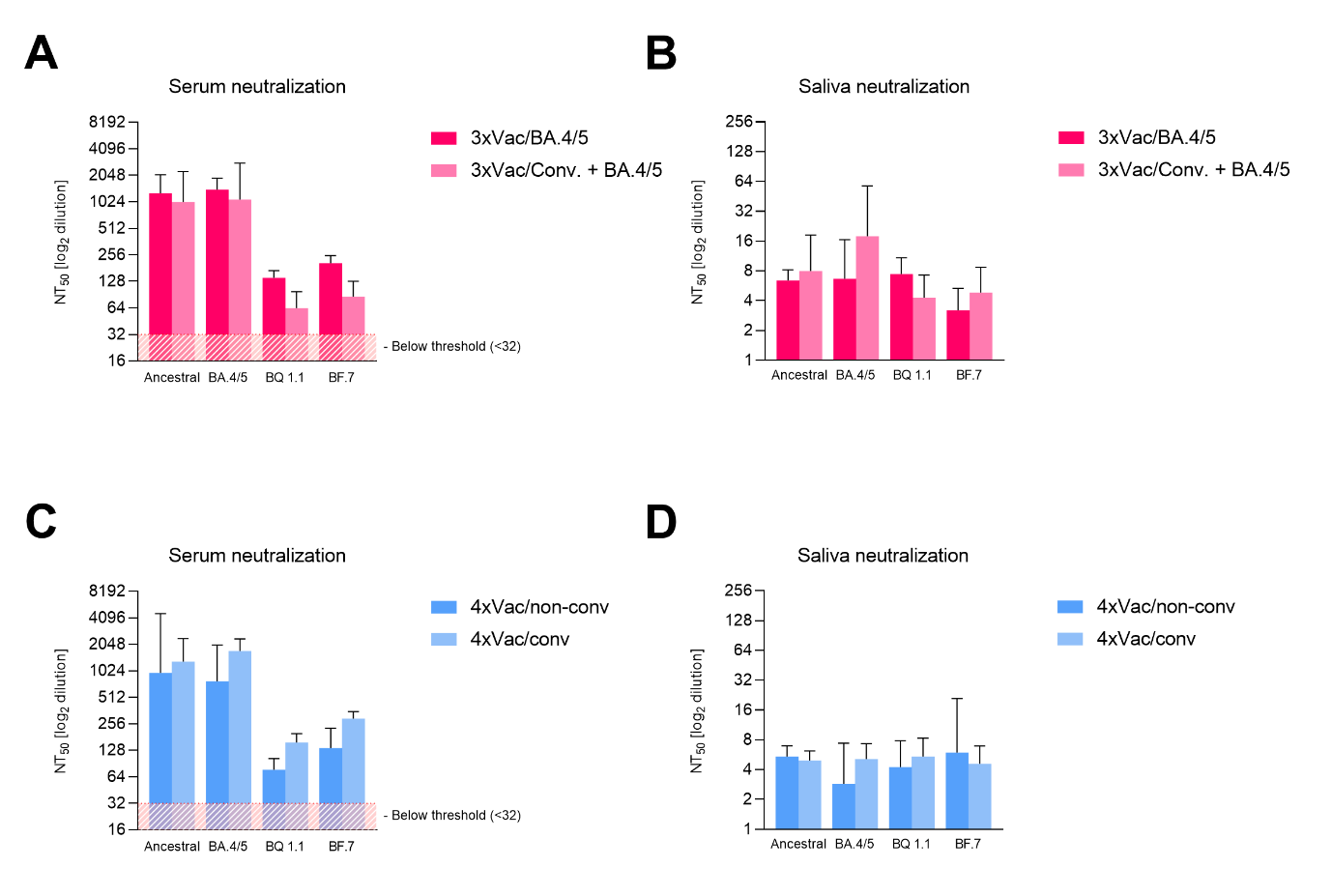


**Figure S5: Differences in viral neutralization of serum and saliva against SARS-CoV-2 variants in cohorts with and without previous infections**

Bar graphs in pink show the half-maximal neutralization titers against ancestral SARS-CoV-2 and omicron subvariants BA.4/5, BQ.1.1, and BF.7 in thrice vaccinated and BA.4/5 convalescent without previous infections (3xVac/BA.4/5) or with previous SARS-CoV-2 infections (3xVac/Conv. + BA.4/5) in serum **(A)** and saliva **(B)**. Blue bar graphs show NT_50_ titers against the tested VOCs in the four times vaccinated cohort with previous infection (4xVac/conv) and without (4xVac/non-conv) in serum **(C)** and saliva **(D)**. Red lines indicate the defined neutralization threshold. For serum, NT_50_>32 was defined as positive and borderline between NT_50_ 16 and 32 and for saliva, NT_50_>1 was defined as positive. Data are expressed as geometric mean ± 95% confidence interval. Statistical significance was determined using non-parametric Kruskal-Wallis test with Dunn’s multiple comparison.


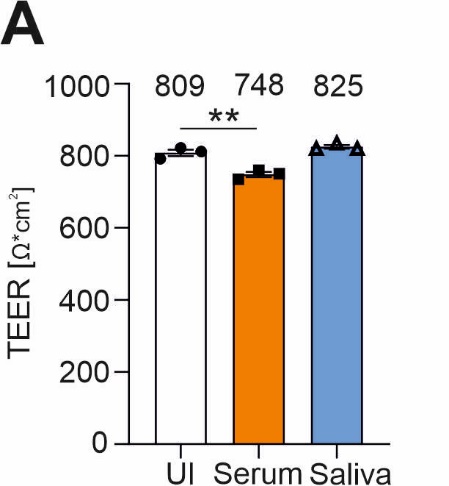


**Figure S6: Tissue integrity after addition of serum and saliva to HAE cells**

TEER from HAE cells where serum (basolateral) or saliva (apical) was added for 72hr or left untreated (UI). All cells were uninfected. Statistically significant differences were determined by one-way ANOVA. The values above each column indicates the mean. Data is presented as mean ± SEM; p<0.01, n=3.

**
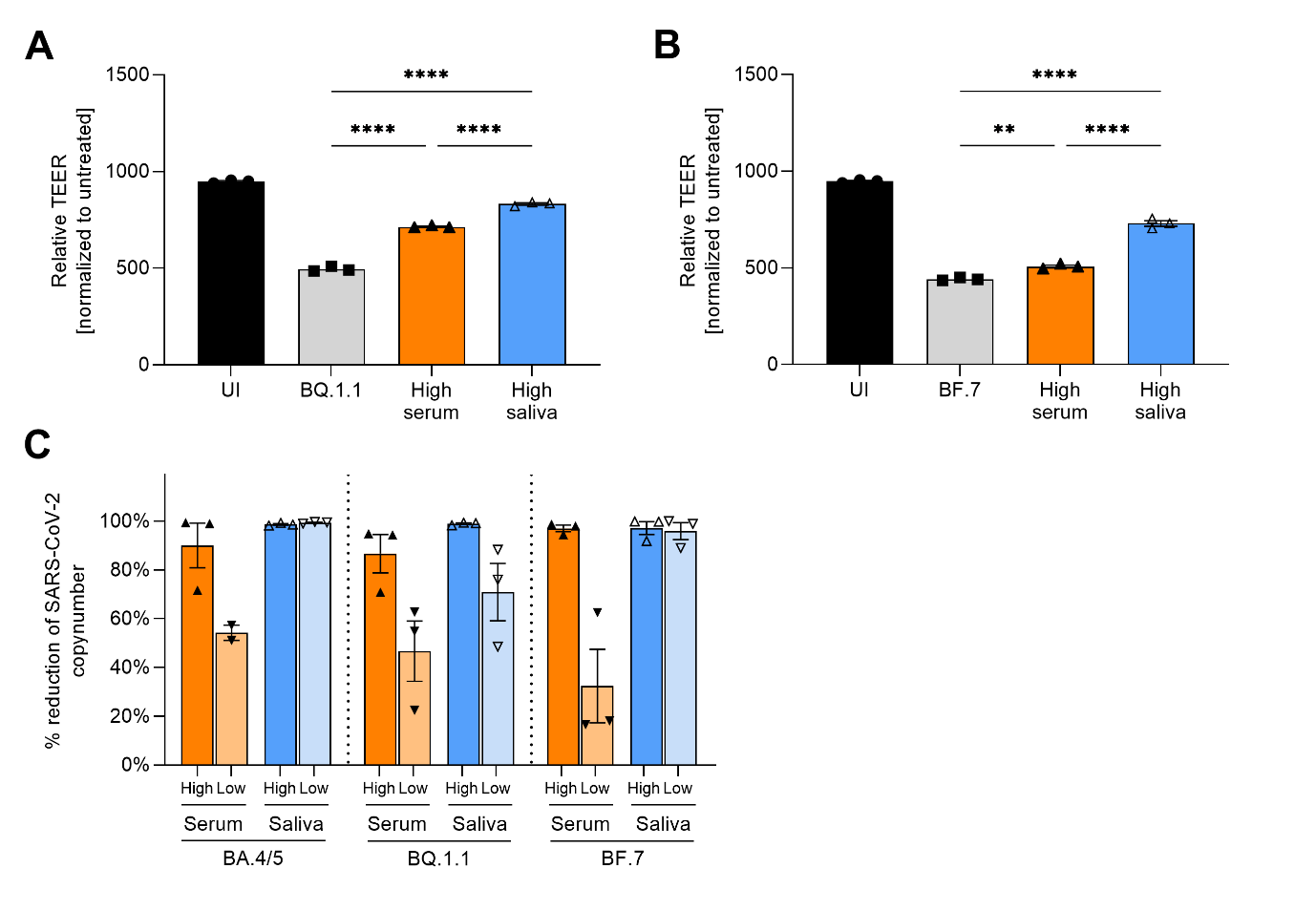
**

**Figure S7: Personalized protection analysis of serum and saliva against Omicron subvariants**

TEER from uninfected (UI) and BQ.1.1 **(A)** or BF.7 **(B)** infected HAE cells from an additional donor was measured in combination with serum and saliva from the high group respectively. Relative reduction of viral load show the differences of serum or saliva from three high or low antibody individuals to protect from SARS-CoV-2 Omicron subvariants BA.4/5, BQ.1.1 or BF.7 compared to infected control **(C)**. Statistically significant differences were determined by one-way ANOVA. Data is presented as mean ± SEM; n=3; p<0.01 (**); p< 0.0001 (****).
